# Occurrence of allergic bronchopulmonary mycosis in patients with asthma: An Eastern India experience

**DOI:** 10.4103/0970-2113.71949

**Published:** 2010

**Authors:** Anirban Sarkar, Abhijit Mukherjee, Aloke Gopal Ghoshal, Somenath Kundu, Subhra Mitra

**Affiliations:** *Department of TB and Respiratory Diseases, Medical College, Kolkata, India*; 1 *State TB Training and Demonstration Centre, Medical College and Hospital*; 2 *Department of TB and Respiratory Diseases, IPGME and R, Kolkata, India*

**Keywords:** ABPA, asthma, central bronchiectasis, Eosinophilia

## Abstract

**Background:**

Allergic bronchopulmonary mycosis (ABPM) is a clinical syndrome associated with immune sensitivity to various fungi notably Aspergillus spp. that colonize the airways of asthmatics. Early diagnosis and treatment with systemic corticosteroids is the key in preventing the progression of the disease to irreversible lung fibrosis.

**Aims:**

To study the occurrence of ABPM among asthma patients with fungal sensitization attending a chest clinic of a tertiary hospital of eastern India. The clinico-radiological and aetiological profiles are also described.

**Materials and Methods:**

All consecutive patients with asthma presenting to the chest clinic over a period of one year were screened for cutaneous hypersensitivity to 12 common fungal antigens. The skin test positive cases were further evaluated for ABPM using standard criteria.

**Results:**

One hundred and twenty-six asthma patients were screened using twelve common fungal antigens; forty patients (31.74%) were found to be skin test positive, and ABPM was diagnosed in ten patients (7.93%). Of the 10 cases of ABPM, nine cases were those of allergic bronchopulmonary aspergillosis (ABPA) and one case was identified as caused by sensitization to Penicillium spp. A majority of the cases of ABPM had advanced disease and had significantly lower FEV1 compared to non-ABPM skin test positive asthmatics. Central bronchiectasis on high resolution CT scan was the most sensitive and specific among the diagnostic parameters.

**Conclusion:**

There is a significant prevalence of ABPM in asthma patients attending our hospital and this reinforces the need to screen asthma patients for fungal sensitisation. This will help in early diagnosis and prevention of irreversible lung damage.

## INTRODUCTION

Allergic bronchopulmonary mycosis (ABPM) is a group of immune-mediated pulmonary diseases that complicates asthma and cystic fibrosis and is caused by colonization of the respiratory tract by various fungi. ABPA entails hypersensitivity to Aspergillus spp., and this entity comprises the majority of cases of ABPM. Because of the indolent nature of the disease, a high index of suspicion is required for an early diagnosis of ABPM. This early diagnosis and subsequent treatment with corticosteroids is the key in preventing the progression of the disease to steroid dependent asthma (stage IV) or fibrotic lung disease (stage V). Increased awareness about this condition among physicians can lead to the diagnosis of this potentially crippling disease at a sufficiently early stage when treatment can save patients from succumbing to end stage lung disease.[1,2]

Since its first recognition in England in1952 and first case reports from India in 1971, there have been quite a few case reports and series studies of ABPA from different parts of the country,[3] yet few or none has been reported from eastern India. This seems to be more due to lack of awareness rather than a lack of incidence. It is in this perspective that this study has been carried out.

## MATERIALS AND METHODS

All consecutive patients of asthma attending the outpatient department of Respiratory Medicine at IPGME and R, Kolkata, over a one-year period were selected for the study. Patients with history of smoking, previous history of tuberculosis, or pregnancy were excluded from the study. Patients currently on oral glucocorticoids or those who have been treated with the same for more than three weeks within the last six months were also excluded. Approval was obtained from our institute ethics committee and written informed consent was procured from all patients.

All patients underwent spirometry and bronchodilator reversibility testing (Recorders and Medicare System: RMS Medspiror machine) and were categorized as having mild, moderate or severe obstructive disease based on their prebronchodilator FEV1 according to the American Thoracic Society (ATS) guidelines.[4] Reversibility was defined as a 12% and 200 ml increase in the FEV1[4] following bronchodilator (400 *μ*g of inhaled Salbutamol).

All selected asthma patients underwent skin prick test (SPT) for 12 common fungal antigens (Creative Drug Industries, Bombay), namely, Aspergillus fumigatus, Aspergillus flavus, Aspergillus niger, Aspergillus tamari, Alterneria alternata, Cladosporum herbarum, Curvularia lunata, Penicillium sp., Fusirium solari, Rhizopas nigricans, Candida albicans, Phoma tropicallis. The result of the test was measured according to Shivpuri’s criteria[5] of grading positive skin prick reactions as follows: negative if wheal is the same as its negative control, doubtful if wheal < 1 ^+^, 1^+^ if wheal is >2 mm than the size of the negative control, 2^+^ if wheal is >4 mm than the size of the negative control, 3^+^ if wheal is >6 mm than the size of the negative control, with or without one or two small pseudopodes, and 4 ^+^ if wheal is >8 mm than the size of the negative control with several pseudopodes.

Only those asthma patients showing positive response to the skin prick test with fungal antigen were investigated further for the diagnosis of ABPA / ABPM. The diagnosis was made when the patient fulfilled either all or four of the five minimal essential criteria [Table 1].[1] Clinical evaluation was done with emphasis on the duration of illness, therapy received and nature of asthma control.

**Table 1:** Criteria for the diagnosis of ABPA in patients with asthma.[1]

Diagnostic criteria	Minimal essential criteria
Asthma	Yes
Central bronchiectasis (inner two thirds of chest CT field)	Yes
Immediate cutaneous reactivity to *Aspergillus* species or *A fumigatus*	Yes
Total serum IgE concentration >417 kU/L (1000 ng/mL)	Yes
Elevated serum IgE– *A fumigatus* and or IgG *-A fumigatus*	Yes
Chest roentgenographic infiltrates	No
Serum precipitating antibodies to *A fumigatus*	No

The peripheral blood eosinophil count was carried out by standard H and E staining - an eosinophil count greater than 350 per cubic mm was taken as eosinophilia.[6]

The total serum IgE was assessed by the ELISA (Premier Medical Corporation, USA) method. Serum specific IgE was determined by ELISA method (Creative Drug Industries, Bombay) against six fungal antigens, namely, Aspergillus fumigatus, Aspergillus flavus, Aspergillus niger, Aspergillus tamari, Alterneria alternate and Penicillium spp. The result was expressed semi quantitatively in grades between 0 and 4^+^.

Serum precipitin tests and specific serum IgGs could not be done due to logistic reasons.

## RADIOLOGY

All recent and previous chest radiographs were reviewed by a radiologist who was not aware of the clinical evaluations or laboratory investigations, for the presence of fleeting opacities in serial chest radiographs, toothpaste or gloved finger shadows indicative of mucus impaction, ring shadows or tramline shadows indicative of bronchiectasis, or for evidence of fibrosis.

High resolution CT (HRCT) scan of the chest was done to look for any bronchiectasis (deemed to be central if confined to the inner 2/3 of the lung field),[1] parenchymal fibrosis, consolidation, atelectasis and mucous plug.

### Isolation of the fungus

Sputum and/or bronchoalveolar lavage fluid was examined for fungal elements by both smear and culture in all SPT positive asthma cases. For smear examination by direct microscopy, sputum or BAL fluid was homogenized by shaking and then a smear was prepared and stained with KOH (10 to 20%). For culture, the sample was first decontaminated by Petroff’s method. Then 0.5 ml was inoculated in SDA medium (Sabouraud’s Dextrose Agar) and SDCC (Sarbouraud’s Dextrose Chloramphenicol Cyclohexidine) medium and incubated at room temperature for a minimum of one week. Identification was based on the morphology of the fungus and its colony.

### Statistical analysis

Statistical analyses of different parameters were done by Paired ‘t’ test and Wilcoxon rank sum test as applicable.

## RESULTS

Of 126 consecutive patients with asthma, 40 patients (31.74%) showed cutaneous hypersensitivity to fungal antigens. Among these 40 patients with skin prick test (SPT) positive asthma, 10 patients (25%) were subsequently diagnosed to be suffering from ABPM. This was 7.93% of our 126 patients with asthma.

The clinical, radiological and pathological aspects of these 40 SPT positive asthma patients (divided into two groups) are given in Table 2.

**Table 2:** Characteristics of skin test positive asthma (non ABPM and ABPM) patients

Characteristics	ABPM (*N*=10)	Non–ABPM (*N*=30)	Significance
Age (years)			
Mean±SD	33.1±11.7	35.8±13.92	Insignificant
Range	19–56	16–68	
Sex			
Male (%)	6 (60)	18 (60)	No
Female (%)	4 (40)	12 (40)	
Duration of wheezing (months)		
Mean±SD	192±149.77	197.83±153	*P*<0.1(insignificant)
Range	40–480	2–600	
Median	144	186	
FEV1 (% predicted)			
Mean±SD	45.8±10.98	57.73±14.04	*P*<0.01
Range	31–63	32–83	
Absolute eosinophil count		
Mean±SD	2048±1848.39	950.8±1279.14	*P*<0.001
Range	280–5304	162–6900	
Median	1448	542	
Serum IgE			
Mean±SD	1420.59±516.56	270±277.69	*P*<0.0001
Range	580–2115.4	6.5–1010	
Median	1330.5	133	
Infiltration on chest X–ray		
Present	10	11	*P*<0.01
Absent	0	19	
Central bronchiectasis and fibrosis on the high resolution CT scan			
Present	10	0	Highly significant
Absent	0	30	

There was a small but insignificant difference (*P*<0.1) in the duration of wheezing in the two groups, with patients of non-ABPM group having longer duration. It was observed that all the 10 cases of ABPM had received at least one course of antitubercular drugs based on their radiological appearance and 60% of cases were referred to us as non- responding tuberculosis.

Spirometry showed that the mean FEV1 was significantly lower in ABPM patients compared to the non-ABPM group of SPT positive asthma patients (45.8% vs. 57.7%, *P*<0.01).

All 40 patients showed immediate hypersensitivity to one or more Aspergillus antigens; 33 (82.5%) patients were sensitive to Aspergillus fumigatus, 19 to A.niger, 15 to A. flavus and two to A. tamarii. Among the 10 ABPM patients, nine (90%) were sensitive to A. fumigatus, four to A niger, one to Penicillium spp. and all 10 to at least one fungal antigen other than Aspergillus. Thirty-nine patients (97.5%) showed sensitivity to at least one of the eight fungal antigens other than the Aspergillus antigens.

Serum total IgE level of more than 1000ng/ml was present in eight of the 10 ABPM patients while this level was found in only one of the 30 non -ABPM patients. Mean serum IgE level was significantly higher in ABPM patients compared to the other group of SPT positive asthma patients (1420.59 vs. 270, *P*<0.0001).

Nine of the 10 ABPM patients had specific IgE against A fumigatus (scale 4), and one had raised (scale 3) specific IgE against Penicillum spp. None of the non-ABPM patients had specific IgE score >1 to the fungal antigens.

Absolute eosinophil count was also seen to be significantly higher in ABPM patients than in non-ABPM SPT positive patients (2048 vs. 950.8, *P*<0.001).

**Figure 1:**
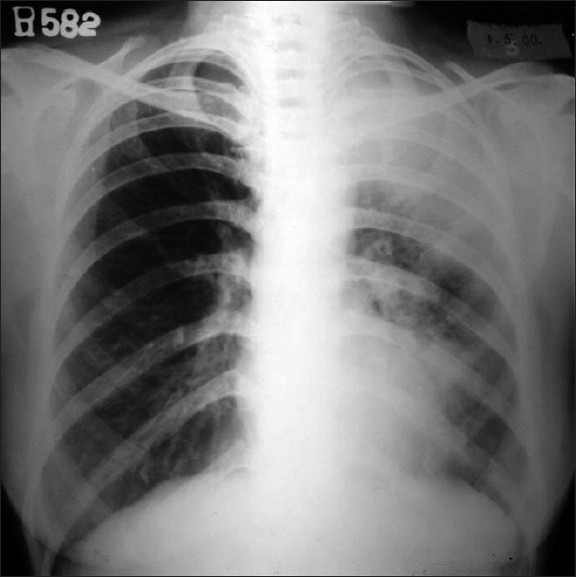
Chest X-ray of a 56-years-old lady with an acute exacerbation of asthma showing left upper lobe collapse-consolidation along with left paracardiac opacity

**Figure 2:**
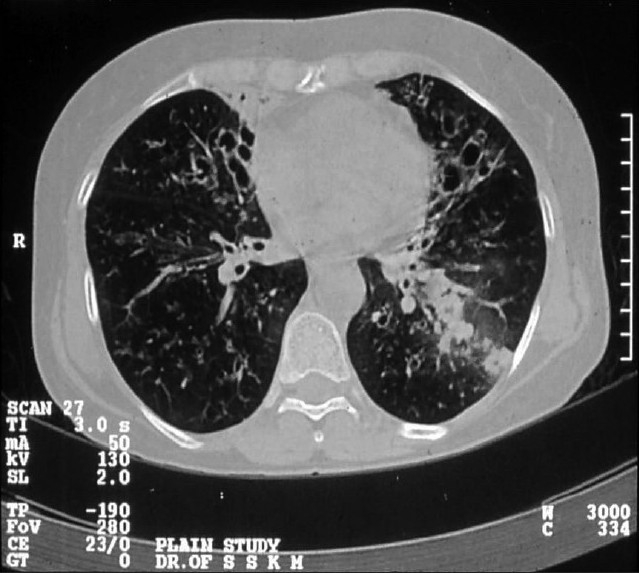
HRCT thorax showing central bronchiectasis

Evaluation of the radiological features revealed that X-ray was normal in 63.3% of non-ABPM patients while all patients with ABPM showed infiltration on the chest radiograph. X-ray opacities included fleeting pattern (50%) and classic gloved finger appearance (20%). High resolution CT scan did not show the characteristic central bronchiectasis with fibrosis in any of the non-ABPM patients while all patients with ABPM had the typical radiological finding. Upper lobe predilection was in 70% cases. These findings were also statistically significant. Figures 1–2 illustrate the characteristic chest X-ray and HRCT pattern of a case of ABPM.

The sensitivity and specificity of the different diagnostic parameters are shown in Table 3.

**Table 3:** Sensitivity and specificity of the different parameters in the diagnosis of ABPA

Diagnostic criteria	Sensitivity	Specificity
Eosinophil count > 350/ mm^3^	90	26.6
Eosinophil count >1000/mm^3^	50	66.6
Serum IgE > 1000 ng/ml	80	96.6
Infiltrate on CXR	100	63.3
Central bronchiectasis on CT scans	100	100
Isolation of fungus	30	80

Figures in parenthesis are in percentage

Five patients of ABPM in our study presented with features of exacerbation stage (stage III), four patients with corticosteroid-dependent asthma (stage IV) and only one patient fulfilled the clinical and radiological features of the end/fibrotic stage (stage V) of the disease.

## DISCUSSION

In an asthma patient, an occurrence of a significant eosinophillia or unexpected infiltrates in the chest X-ray raises the suspicion of ABPA making it important to determine if the patient is SPT positive to Aspergillus or other relevant fungal antigens. A positive SPT should prompt evaluation for the diagnosis of ABPA or other rarer examples of ABPM such as those caused by Penicillum, Candida, Curvularia or Helminthosporium spp.

Among asthmatics various studies show SPT positivity to Aspergillus ranging from 14 to 46%.[7–11] In India Maurya *et al*[12] and Agarwal *et al*,[2] reported Aspergillus SPT positivity in 28.5 and 39.5% of studied patients, respectively. In our study skin test positivity to Aspergillus antigens was found in 31.75% of the asthma patients.

The prevalence of ABPA among asthma patients vary given the lack of uniform diagnostic criteria and standardized tests.[3] Greenberger *et al*,[13,14] suggested that ABPA could complicate 1-2% of all cases of asthma. Eaton *et al*,[7] argued that the prevalence of ABPA in a typical asthma clinic was likely to exceed 5%. Kumar and Gaur[15] reported 16% ABPA prevalence in their asthma subjects, while Maurya *et al*,[12] reported an ABPA prevalence of 7.5% in their asthma subjects from Delhi and Agarwal *et al*,[2] reported an ABPA prevalence as high as 27.5% in their study of 564 asthma patients from north India. Such high figures probably indicate a referral bias. In the present study, the prevalence of ABPM among asthma subjects in a tertiary care referral hospital in eastern India was 7.93% in one year, which represent about 25% of SPT positive asthmatics. Further, our study shows that 90% cases of ABPM were due to A. fumigatus, which corroborates the fact that aspergillus is the most common pathogen of ABPM.

Greenberger *et al*,[16] reported a history of asthma being present for more than 10 years before the diagnosis of ABPA. Schwartz *et al*,[6] observed a trend toward more severe airway obstruction in ABPA patients. Seventy percent of our ABPM cases had h/o asthma for more than 10 years and 50% had moderately severe airway obstruction (FEV1 41-60% predicted). For the diagnosis of ABPA, five minimal criteria have been proposed in a recent review by Greenberger[1] [Table 1]. Of these, asthma, positive skin test with A. fumigatus and central bronchiectasis on CT scan had been considered as the "minimal essential" criteria in a previous study by Schwartz HJ *et al*.[6] Out of the 10 patients of ABPM, eight patients showed positive results with all the five criteria. In two patients total serum Ig E concentration was less than 1000ng/ml.

Serum total IgE > 1000ng/ml, although a typical finding in ABPA patients,[17] is neither sensitive nor specific for the diagnosis of ABPA as lower levels are expected in patients with fibrotic end stage disease, in patients in remission (stage II) and in patients on oral corticosteroids.[7] Of the two patients who had serum IgE levels less than 1000 ng/ml, one was on oral corticosteroid for the relief of asthma and the other had end-stage disease.

Eosinophil count has neither proved to be sensitive nor specific for the diagnosis of ABPA in previous studies as well as in the present one.

Central bronchiectasis is the most important radiological finding in ABPA and is almost pathognomic of ABPA in the absence of cystic fibrosis. The routine use of HRCT scans in skin test positive asthmatics allows central bronchiectasis to be diagnosed earlier and with more precision.[18–20]

In our study, all patients of SPT positive asthma underwent HRCT thorax. The finding of central bronchiectasis in HRCT was both 100% sensitive and 100% specific in the diagnosis of ABPM. CT scanning offered the explanation for radiological findings in non-ABPM SPT positive asthma patients. Also CT scan helped in the identification of two additional cases of ABPM who did not have raised serum IgE.

The rate of finding positive sputum culture in ABPA varies from 82.6%,[21] 60%[22] to 15.3%[8] in the different Indian studies. In the study by Shivananda,[8] A. fumigatus was the most commonly isolated Aspergillus species (11.5%), followed by A. niger (3.2%) and A. flavus (0.9%).

In the present study [Table 4], in three out of six cases of ABPM where fungus was isolated from sputum, it was Candida sp. which represented oropharyngeal colonization as bronchoalveolar lavage (BAL) fluid did not grow any organism. In the other three cases, both sputum and BAL fluid grew A. fumigatus (2 cases) and Penicillium. Among SPT positive asthma, non- ABPM patients, both sputum and BAL fluid grew Candida sp. in three cases, A. fumigatus in one case and A. niger in two cases. Thus sensitivity and specificity of fungus isolation in the diagnosis of ABPM were 30 (3/10) and 80% (24/30), respectively

**Table 4:** Fungus isolated from sputum and BAL

Fungus isolated	Non–ABPM	ABPM
Candida sp.	3	0
A. fumigatus	1	2
A. niger	2	0
Penicillium	0	1
None	24	7
Total	30	10

Although there have been case reports of ABPM other than ABPA, it remains likely that ABPM, other than ABPA, is rare as in the opinion of Greenberger.[22] In our study, of the ten patients of ABPM, only one case showed strong skin test positive reaction to Penicillium sp.; specific IgE to Penicillum was positive (3^+^) and the sputum and bronchoalveolar lavage fluid consistently grew a pure culture of Penicillium. This case is considered as a case of ABPM, caused by Penicillium sp.

ABPA has been staged[1,23] as stage I (acute), stage II (remission), stage III (exacerbation), stage IV (corticosteroid-dependent asthma), stage V (fibrotic). In our series 50% patients were in stage III followed by 40% in stage IV and 10% in stage V. Routine screening of all asthma patients for ABPM is expected to detect patients at an earlier stage.

To conclude, a diligent search for ABPM should be considered in all cases of mould-sensitive asthma and HRCT scan of thorax should probably be included as a part of the diagnostic algorithm. Increased awareness of this potentially crippling disease is essential to make an early diagnosis and to institute appropriate therapy in order to prevent irreversible lung damage.
